# More than 1000 genotypes are required to derive robust relationships between yield, yield stability and physiological parameters: a computational study on wheat crop

**DOI:** 10.1007/s00122-023-04264-7

**Published:** 2023-03-10

**Authors:** Tien-Cheng Wang, Pierre Casadebaig, Tsu-Wei Chen

**Affiliations:** 1 Present Address: Section of Intensive Plant Food Systems, Albrecht Daniel Thaer-Institute of Agricultural and Horticultural Sciences, Humboldt Universität zu Berlin, Berlin, Germany; 2 Institut für Gartenbauliche Produktionssysteme, Leibniz Universität Hannover, Hannover, Germany; 3 INRAE, UMR AGIR, Université de Toulouse, 31320 Castanet-Tolosan, France

## Abstract

**Key message:**

Using in silico experiment in crop model, we identified different physiological regulations of yield and yield stability, as well as quantify the genotype and environment numbers required for analysing yield stability convincingly.

**Abstract:**

Identifying target traits for breeding stable and high-yielded cultivars simultaneously is difficult due to limited knowledge of physiological mechanisms behind yield stability. Besides, there is no consensus about the adequacy of a stability index (SI) and the minimal number of environments and genotypes required for evaluating yield stability. We studied this question using the crop model APSIM-Wheat to simulate 9100 virtual genotypes grown under 9000 environments. By analysing the simulated data, we showed that the shape of phenotype distributions affected the correlation between SI and mean yield and the genotypic superiority measure (*P*_i_) was least affected among 11 SI. *P*_i_ was used as index to demonstrate that more than 150 environments were required to estimate yield stability of a genotype convincingly and more than 1000 genotypes were necessary to evaluate the contribution of a physiological parameter to yield stability. Network analyses suggested that a physiological parameter contributed preferentially to yield or *P*_i_. For example, soil water absorption efficiency and potential grain filling rate explained better the variations in yield than in *P*_i_; while light extinction coefficient and radiation use efficiency were more correlated with *P*_i_ than with yield. The high number of genotypes and environments required for studying *P*_i_ highlight the necessity and potential of in silico experiments to better understand the mechanisms behind yield stability.

**Supplementary Information:**

The online version contains supplementary material available at 10.1007/s00122-023-04264-7.

## Introduction

To ensure global food security, it is not only important to increase yield gain but also yield stability. Developing stable crop cultivars is therefore crucial maintaining the yield level and adapting to ever-changing weather schemes (Powell et al. 2012; Dwivedi et al. 2016; Macholdt and Honermeier 2017; Bocci et al. 2020; van Frank et al. 2020). Breeding stable plants requires profound crop physiological knowledge and empirical experiences to identify target traits. However, our physiological understanding of yield stability is still scarce (Pedro et al. 2011) since assessing the yield stability of a genotype requires field experiments across multiple years, locations, agriculture practices and comparisons with other genotypes (e.g. 440 progenies in 16 environments in Wang et al. 2015; 191 cultivars under 43 environments in Voss-Fels et al. 2019; 720 lines in 36 environments in Sehgal et al. 2017). Therefore, identifying cultivars with stable yields is time-consuming and labour intensive, which significantly restricts the speed of our knowledge gain in the eco-physiological mechanisms and their genetic controls resulting in yield stability. Furthermore, there is no consensus in the literature about (1) the adequacy of a stability index (SI) to quantify yield stability and (2) the minimal number of sampled environments and sampled genotypes required for evaluating the yield stability (Reckling et al. 2021). In other words, the minimal size of sampled populations of genotypes and of environments for assessing yield stability is unclear. Also, if a population of genotypes is selected, it is unknown how the yield stability of an individual genotype in the population is affected by the phenotypic distribution (e.g. yield distribution) of this population.

In the past, breeders discovered performance and stability related-traits based on their physiological knowledge and practical experience in the field (Bolaños and Edmeades 1993; Pfeiffer et al. 2001; Pedro et al. 2011). Nowadays, crop modelling and simulation can complement such empirical knowledge by generating thousands of virtual genotypes by subtle changes in structural and physiological parameters (Chen et al. 2015; Casadebaig et al. 2016; Perez et al. 2018) and ultimately help to identify structural and physiological traits of an ideotype. This approach allows us to quantify the potential contributions of a physiological parameter to the performance of new cultivars in test environments, accounting for a large climatic variability, the so-called target population of environments (Quilot-Turion et al. 2012; Senapati and Semenov 2020). For example, using the Sirius crop model, stay green and flag leaf area are identified as crucial parameters of wheat (*Triticum aestivum* L.) under drought and heat stress. It suggests a potential to increase the yield of current wheat cultivars in Europe by 3.5–5.2 t ha^−1^ (Senapati and Semenov 2019). Since crop modelling predicts crop performance in response to given management or climatic regimes (Chenu et al. 2011; Barillot et al. 2014; Kouadio et al. 2015; Casadebaig et al. 2016; Sun et al. 2016; Parent et al. 2018; Leakey et al. 2019; Wu et al. 2019), simulated yield data obtained from crop models may be also used to analyse yield stability and to estimate the minimal population size of sampled environments and genotypes required for evaluating yield stability.

Here we reviewed and compared 11 stability indices for their adequacy to inform plant breeding for both crop yield and stability. We first developed an *R* package (with 11 stability indices including static, dynamic, probabilistic, parametric and nonparametric indices; Wang and Chen 2022), which facilitates the study of stability analysis and well-integrated with other packages for further analysis in *R* environment. Second, we reused an  in silico experiment conducted with the APSIM-Wheat crop model to analyse yield performance of 9100 virtual genotypes grown under 9000 environments (Casadebaig et al. 2016). Data from the in silico experiments enabled (1) to demonstrate the analysis pipeline; (2) to determine the minimal number of genotypes and environments to assess yield stability; (3) to identify the contribution of physiological traits on yield stability and (4) to propose physiological mechanisms to achieve stable yield.

## Material and methods

### Dataset obtained from in silico experiment with the APSIM-wheat

Crop model APSIM-Wheat **(**www.apsim.info**)** was used to simulate a dataset (10.5281/zenodo.4729636; for details, see Casadebaig et al. 2016) with 9100 virtual genotypes (*N*_gen_ = 9100) grown under 9000 environments (*N*_env_ = 9000). In short, virtual genotypes were created by varying the value of 90 independent physiological parameters in a range of ± 20% from the reference cultivar *Hartog*, which represents the default parameter values in the APSIM-Wheat. Environments in the dataset contain historical climate data of 125 years (1889–2013) in four locations in Australia (Emerald, Narrabri, Yanco and Merredin, see also Table 1 from Casadebaig et al. 2016) in Australia, in combination with two CO_2_ levels (380 and 555 ppm), three nitrogen levels (low: 50%, control: 100% and high fertilization: 100% plus 50 kg ha^−1^) and three sowing dates (early, control and late). Eight integrated model outputs (grain and straw yields, grain size, grain number, grain protein, leaf area index (LAI), maturity date and flowering date) were used for trait stability analysis. Straw yield was calculated by subtracting grain yield from biomass.

**Table 1:** Mean tendency of a parameter (*T*_parameter_), *|r|* to yield and to *P*_i,yield_ and parameter (*R*^2^ and slope) of linear regression of |*r|* to yield versus |*r|* to *P*_i,yield_ from six physiological parameters from 100 SPG in Fig. 4

Sampling method	Physiological parameter	*T*_*parameter*_	*|r|* to yield	*|r|* to *P*_i,yield_	*R*^2^	Slope
Even	*y_rue*	1.01 ± 0.01	0.76 ± 0.04	0.77 ± 0.03	0.96	0.96
Random	*y_rue*	1.16 ± 0.08	0.39 ± 0.08	0.45 ± 0.08	0.95	0.94
Top 20	*y_rue*	1.55 ± 0.93	0.20 ± 0.09	0.27 ± 0.10	0.72	0.88
Even	*y_sla*	1.01 ± 0.01	0.64 ± 0.05	0.65 ± 0.05	0.98	0.96
Random	*y_sla*	1.28 ± 1.44	0.24 ± 0.10	0.26 ± 0.10	0.94	0.96
Top 20	*y_sla*	1.28 ± 0.77	0.20 ± 0.09	0.22 ± 0.09	0.75	0.91
Even	*ll_modifier*	0.77 ± 0.10	0.27 ± 0.07	0.21 ± 0.07	0.99	1.02
Random	*ll_modifier*	0.82 ± 0.10	0.47 ± 0.09	0.39 ± 0.10	0.91	1.11
Top 20	*ll_modifier*	8.52 ± 65.02	0.13 ± 0.08	0.10 ± 0.07	0	− 0.05
Even	*potential_grain_filling_rate*	0.98 ± 0.02	0.56 ± 0.06	0.55 ± 0.06	0.98	1.03
Random	*potential_grain_filling_rate*	0.87 ± 0.12	0.29 ± 0.10	0.26 ± 0.10	0.96	0.98
Top 20	*potential_grain_filling_rate*	0.85 ± 0.12	0.48 ± 0.06	0.41 ± 0.08	0.52	0.96
Even	*node_no_correction*	0.78 ± 0.20	0.18 ± 0.06	0.15 ± 0.06	0.96	0.98
Random	*node_no_correction*	0.93 ± 0.90	0.21 ± 0.09	0.18 ± 0.09	0.95	0.96
Top 20	*node_no_correction*	0.67 ± 0.14	0.43 ± 0.07	0.29 ± 0.09	0.63	1.05
Even	*tfac_slope*	1.01 ± 0.03	0.35 ± 0.06	0.35 ± 0.06	0.98	1.02
Random	*tfac_slope*	2.15 ± 6.94	0.09 ± 0.07	0.09 ± 0.07	0.8	0.82
Top 20	*tfac_slope*	1.71 ± 0.59	0.23 ± 0.08	0.37 ± 0.08	0.73	0.82

Meaning of the physiological parameters (in *italic*) follow the order (left to right in Fig. 4): radiation use efficiency (*y_rue*), potential leaf specific area (*y_sla*), efficiency of roots to extract soil water (*ll_modifier*), potential grain growth rate at grain filling (*potential_grain_filling_rate*), number of growing leaves in the sheath (*node_no_correction*) and temperature effect on biomass accumulation (*tfac_slope*).

### Computation of stability indices of the virtual genotypes with three sampling methods

All analyses were implemented in the *R* environment (R Core Team 2020) where 11 stability indices (SI) were calculated in a customized package *toolStability* (Wang and Chen 2022; https://github.com/Illustratien/toolStability). The SI in *toolStability* include static and dynamic concepts of stability (Becker and Léon 1988). Under the static concept, the trait of a stable cultivar stays relatively unchanged across different environments. In contrast, dynamic concept takes the environmental mean into account and considers the interactions between genotypes and environments. Furthermore, each concept can be further classified as parametric or nonparametric. In *toolStability*, there are two parametric SI of static concept: environmental variance (Römer 1917) and adjusted coefficient of variation (Reckling et al. 2018). A dynamic concept has nine SI, eight parametric and one nonparametric. Parametric dynamic SI are: coefficient of determination (Pinthus 1973), coefficient of regression (Finlay and Wilkinson 1963), deviation mean squares (Eberhart and Russell, 1966), ecovalence (Wricke 1962), genotypic stability (Hanson 1970), genotypic superiority measure (Lin and Binns 1988), safety first index (Eskridge 1990) and stability variance (Shukla 1972). Depending on the value, the coefficient of regression can be static or dynamic (Becker and Léon 1988). The only nonparametric SI in *toolStability* is the variance of rank (Nassar and Hühn 1987). Each SI represents a specific way of describing a kind of interaction between genotypes and environments. The choice of SI depends on the research question. In this study, we focus on the SI that highly correlates with the genotypic mean yield from all environments to target high and stable yield for crop breeding.

To be consistent between the dimensions of trait (e.g. yield, t ha^−1^) and stability indices, indices which with squared units of trait were square-rooted to avoid artificial nonlinear relationship between trait and SI (e.g. genotypic superiority index, *P*_i_, Lin and Binns 1988, ecovalence, *W*_i,_ Wricke 1962 and variance of rank, *S*_i_4, Nassar and Hühn 1987). We noticed that the value of ecovalence (*W*_i_) depends on the number of environments. To ensure the comparability of *W*_i_ between calculations with different number of environments, a modified ecovalence (*W*_i_^‘^) is proposed as dividing original ecovalence (*W*_i_) with the number of environments. The dimension-less indices remained unmodified (e.g. *b*_i_, Finlay and Wilkinson 1963).

To calculate a SI of a genotype, data of multiple genotypes (referred to as sampled population of genotypes, SPG, selected from 9100 genotypes pool) grown under multiple environments (referred to as sampled population of environments, SPE, selected from 9000 environments pool) are required. If a SI is highly correlated with the mean yield from all environments in the studied genotypes, this SI indicates a high and stable yield at the same time. As a first step, we investigated whether the shape of phenotypic distribution (e.g. yield distribution in the SPG) affects the relationship between mean yield and SI (Fig. S1k). For this, three sampling methods were used: (1) “*random*” sampling method that resulted in a population with normal distribution, which is commonly found in real breeding programs (Powell and Rutten 2013); (2) “*even*” sampling method with a flat and even distributed population (Breseghello et al. 2009), which is created by first dividing the whole population into ten deciles based on the mean genotypic yield in 9000 environments and then randomly sampled 10% of total sampling number in each decile; and 3) “*top 20*” method representing the population of elite cultivars that had yield values larger than 80% of genotypes from the whole population (Longin and Reif 2014). For each sampling method, 100 virtual genotypes (number of genotypes in each SPG, *N*_gen_ = 100) grown under 100 environments (number of randomly selected environments in each SPE, *N*_env_ = 100) were first selected to test how the shape of phenotype distribution affects the relationship between mean yield and 11 SI.

### Estimating the minimal number of environments required to estimate yield stability

To represent both high and stable performance of yield, genotypic superiority measure of yield (*P*_i,yield_) was selected to demonstrate the minimal required *N*_env_ for reliable estimation of a SI. We first calculated *P*_i,yield_ of 100 virtual genotypes (*N*_gen_ = 100) in 100 SPE, in combination with different *N*_env_ ranging from 3–600 using “*random*” sampling method. Secondly, the coefficient of variation of *P*_i,yield_ (CV_*P*i,yield_) was calculated for each *N*_env_ between 100 SPG. An arbitrary low threshold value (i.e. 5 or 10% CV_*P*i,yield_, Piepho 1998) was used to determine the minimal required *N*_env_ to estimate yield stability. Moreover, to test the effect of sampling methods on CV_SI,yield_ of other 10 SI, the same setting (*N*_gen_ = 100, *N*_env_ = 10–600 and SPE = 100) was applied.

### Analysis of the correlation network between plant traits, crop performance and stability

A network analysis (node and edge graph) was performed to illustrate the Pearson correlation coefficient (*r*, referred to edge in a network) between yield, *P*_i,yield_ and physiological parameters (referred to node) of genotypes (*N*_gen_ = 100, SPG = 100) selected by three sampling methods (i.e. “*random*”, “*even*” and “*top 20*”) in a SPE. For each SPG, a table listing mean yield, *P*_i,yield_ and 90 physiological parameters of each genotype was created (Supplementary Fig. S2a). The Pearson correlation coefficients between all columns in this table were calculated to produce a *r*-matrix (Supplementary Fig. S2b), which was further transformed into a linear vector format (*r*-vector, Supplementary Fig. S2c) with 181 values (total number of all combinations, ${C}_{2}^{92}$=4186, minus number of correlations between physiological parameters, ${C}_{2}^{90}$=4005). To identify whether a physiological parameter tends to explain yield or *P*_i,yield_ more, a tendency index (*T*_parameter_) in each SPG was quantified by the ratio of absolute *r* value (|*r*|) for *P*_i,yield_ (|*r*| between *P*_i,yield_ and physiological parameter) to |*r*| for yield (|*r*| between yield and physiological parameter). If *T*_parameter_ > 1, this parameter is more related to *P*_i,yield_ than yield. On the other hand, a parameter explains yield more than *P*_i,yield_ when *T*_parameter_ < 1.

### Estimating the minimum number of genotypes for robust correlation networks between plant traits, crop performance and stability

To acquire the minimum *N*_gen_ and *N*_env_ that produce the robust and representative correlation between yield, *P*_i,yield_ and physiological parameters, we evaluated the overall strength of correlation network by four steps: (1) 100 SPG in combination with nine genotype numbers (*N*_gen_ = 5, 50, 100, 200, 300, 500, 700, 900 and 1100) and six environment numbers (*N*_env_ = 5, 50, 100, 300, 500 and 700, SPE = 1) were sampled to obtain 5400 *r*-vectors (Supplementary Fig. S2d); (2) a table listing *r*-vectors of 100 SPG was created for each combination of genotype and environment numbers (Supplementary Fig. S2e); (3) since the similarity between different *r*-networks can be represented by calculating *r* between two *r*-vectors in this table, an “edge-*r*-matrix” representing *r* between 100 *r*-vectors from 100 SPG in this table was calculated (Supplementary Fig. S2f); (4) the edge-*r*-matrix was squared (to represent power of explanation for correlation between nodes) and averaged to obtain an indicator *S* representing the similarity between networks of 100 SPG (Supplementary Fig. S2g). If *S* is close to one, networks between SPG are similar and if *S* = 0, networks between SPG are completely different.

## Results

### Relationships between mean yield and yield stability were affected by the sampling methods.

To facilitate and reproduce our yield stability analysis, we developed “*toolStability*”, which is an *R* package (Wang and Chen 2022) available on a public repository providing a wide range of functions to calculate 11 stability indices (SI). From the 9100 virtual genotypes created by the APSIM-Wheat, 100 of them were selected (number of genotypes, *N*_gen_ = 100) by three sampling methods (i.e. “*random*”, “*even*” and “*top 20*”) for 100 times (sampled population of genotype, SPG = 100) in 100 environments (number of environments, *N*_env_ = 100, sampled population of environment, SPE = 1), resulting in different shape of phenotype distributions (Supplementary Fig. S1k) which represent different strategies or steps in the breeding program.

**Fig. 1:**
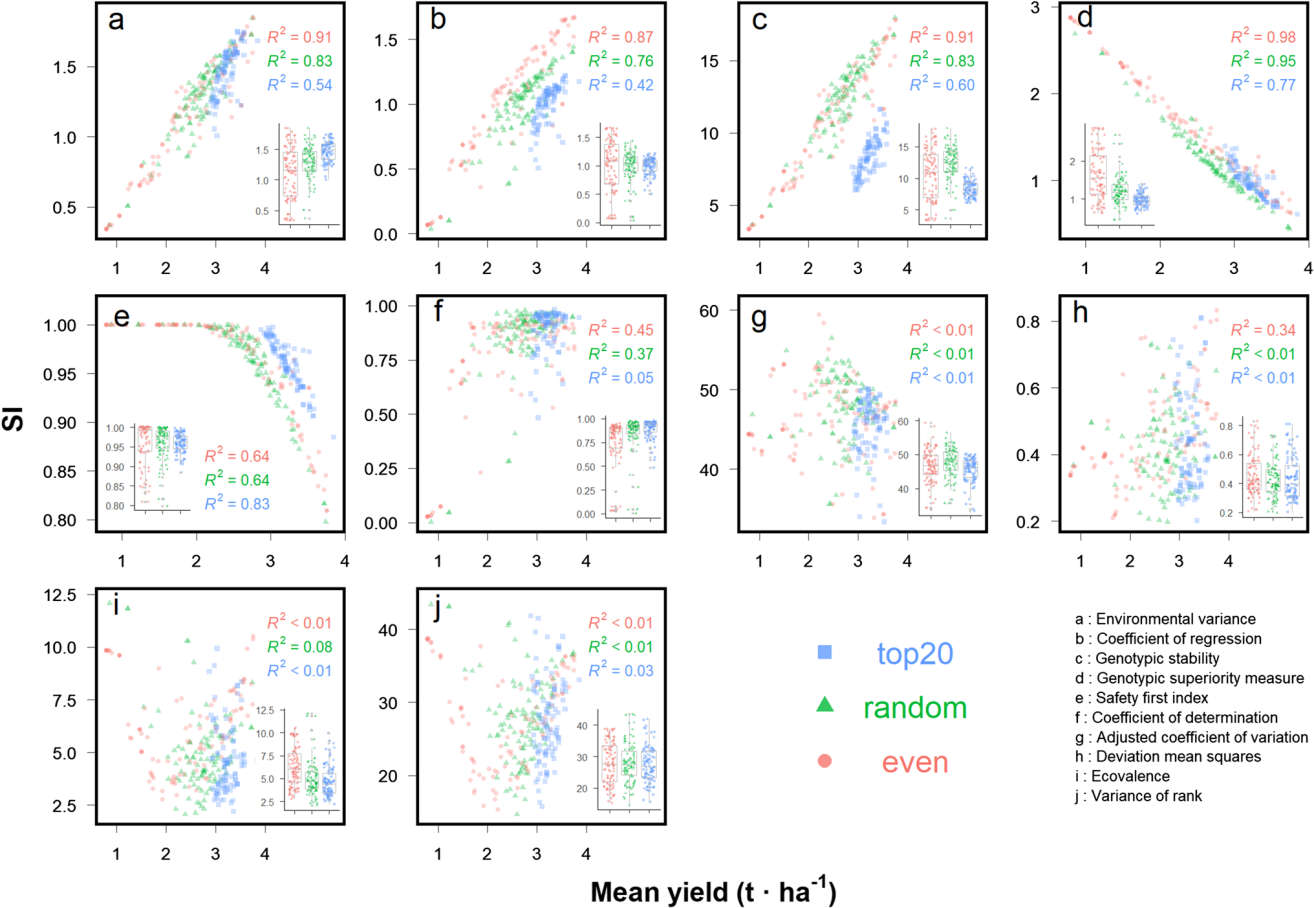
Comparison of relationship between mean yield and ten stability indices (SI) of randomly selected 100 virtual genotypes (SPG = 1) under 100 environments (SPE = 1) with three sampling methods. Each point represents a genotype. Red, green and blue colours indicate sampling method “*even*”, “*random*” and “*top 20*”, respectively. Subfigures show the boxplots of SI. The “*even*” sampling method divides population into ten groups based on the mean yield value, with ten virtual genotypes randomly selected in each yield group. The “*top 20*” method randomly selects virtual genotypes with mean yield above 80th percentile of whole population**.** Ten SI are (**a**) environmental variance, **b** coefficient of regression, **c** genotypic stability, **d** genotypic superiority measure, **e** safety first index, **f** coefficient of determination, **g** adjusted coefficient of variation, **h** deviation mean squares, **i** ecovalence and **j** variance of rank (colour figure online)

Pearson correlation coefficient (*r*) between trait (e.g. mean yield) and SI was used to identify the SI that represent stable and high trait performance simultaneously. Three SI correlated positively to mean yield (Fig. 1a–c): environmental variance (*W*_i,yield_), coefficient of regression (*b*_i,yield_) and genotypic stability (*D*^*2*^_i,yield_). Two SI negatively correlated with mean yield (Fig. 1d–e): genotypic superiority measure (*P*_i,yield_) and safety first index. Other five SI showed low correlations (*R*^*2*^ < 0.5) with mean yield and were not suitable for selecting high and stable yield at the same time (Fig. 1f–j). Low correlation between *W*_i,yield_ and genotypic mean yield (Fig. 1i,* R*^2^ < 0.08) was expected due to the orthogonal relationship between genotypic mean yield and the effect of interaction of genotype by environment (Mohammadi and Amri 2008). Another SI, *S*_i_4_,yield_, was highly correlated with *W*_i,yield_ and expected to have also low correlation (Fig. 1j *R*^2^ < 0.03) to genotypic mean yield, as reported in the literature (Piepho and Lotito 1992).

Sampling method affected the correlation between SI and mean yield (Fig. 1) and the shape of SI distribution (Supplementary Fig. S1). In general, the ranking of *R*^2^ between mean yield and four SI was the highest in “*even*” selection method, followed by “*random*” selection and the lowest in “*top 20*” selection method. Taking *b*_i,yield_ (Fig. 1c) as example, the effect of sampling method on correlation between mean yield and *b*_i,yield_ was the highest in “*even*” (*R*^2^ = 0.87), followed by “*random*” (*R*^2^ = 0.76) and the lowest in “*top 20*” (*R*^2^ = 0.42). Among the 11 studied SI, the linear correlation between *P*_i,yield_ and mean yield was least affected by the sampling methods (Fig. 1d), with *R*^2^ of 0.98, 0.95 and 0.77 in methods “*even*,” “*random*” and “*top 20*”, respectively. This is also a reason to use *P*_i_ as the representative stability index in the following analysis in this study.

### More than 150 environments are required to estimate genotypic yield stability robustly using 100 genotypes

**Fig. 2:**
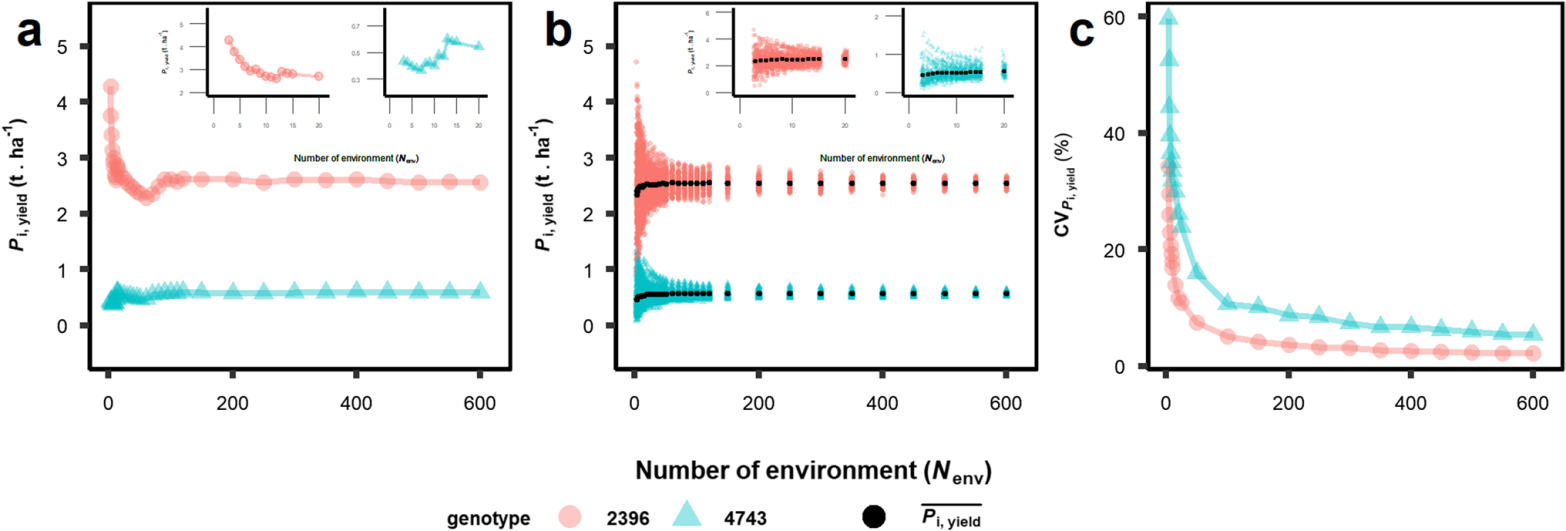
Relationships between *P*_i,yield_ and environment number (*N*_env_, sampled with 100 virtual genotypes). Two genotypes with contrasting *P*_i,yield_ values were chosen: red circle for unstable genotype 2396 and blue triangle for stable genotype 4743. **a** Results from single sampled population of environment (SPE = 1). **b** Results from SPE = 100. Black circles stand for the averaged *P*_i,yield_ value from 100 SPE. **c** Coefficient of variation of *P*_i,yield_ (CV_*P*i,yield_) between 100 SPE (colour figure online)

To test the minimum *N*_env_ required for robust estimation of *P*_i,yield_ of a genotype, 100 random virtual genotypes (*N*_gen_ = 100, SPG = 1) created by APSIM-Wheat were first selected by “*random*” method, then their yields were simulated from 3 to 600 random environments (*N*_env_ = 3–600). The selections of environments were repeated 100 times (sampled population of environments, SPE = 100), and *P*_i,yield_ of genotypes in each SPE was calculated. Within one SPE (Fig. 2a), the range of *P*_i,yield_ of an unstable genotype (represented by genotype 2396) between different *N*_env_ varied from 2.28 to 4.28 t ha^−1^, with coefficient of variation (CV) of 14.1%. In comparison with genotype 2396, a stable genotype (represented by genotype number 4743) under the same SPE had similar CV of *P*_i,yield_ (14.5%) but a smaller range of *P*_i,yield_ (from 0.36 to 0.6 t ha^−1^). Irregular variations in *P*_i,yield_ in both genotypes (subfigures in Fig. 2a) indicated strong effects of SPE on the estimation of *P*_i,yield_. Using 100 SPE, the potential bias of estimated *P*_i,yield_ from the sampling of environments were quantified (Fig. 2b). *P*_i,yield_ estimated from 100 SPE with three environments (*N*_env_ = 3) varied largely in stable and unstable genotypes (0.08–1.56 and 0.47–4.73 t ha^−1^, respectively), indicating unreliable estimation of *P*_i,yield_ at low *N*_env_. Standard deviation of *P*_i,yield_ between 100 SPE decreased with the increase of *N*_env_, while the mean of *P*_i,yield_ from 100 SPE increased with *N*_env_ slightly in an asymptotic manner.

Based on the results shown in Fig. 2b, coefficient of variation (CV_*P*i,yield_) between SPE was calculated for each *N*_env_ (Fig. 2c). The minimal *N*_env_ was defined as *N*_env_ at which CV_*P*i,yield_ became lower than the predefined thresholds (5 or 10%). Stable genotype 4743 was found with larger CV_*P*i,yield_ than unstable genotype 2396. The relationship between CV_*P*i,yield_ and *N*_env_ was fitted by an exponential function CV_*P*i,yield_ = α*N_env_^*β*^, where α adjusts the range of CV_*P*i,yield_ and *β* controls the curvature. For a stable genotype 4743, CV_*P*i,yield_ = 0.93 * *N*_env_^−0.44^ (*R*^2^ = 0.993, *p*-value < 0.001, se_*α*_ = 0.06, se_*β*_ = 0.012). For an unstable genotype 2396, CV_*P*i,yield_ = 0.52 * *N*_env_^−0.50^ (*R*^2^ = 0.999, *p*-value < 0.001, se_*α*_ = 0.03, se_*β*_ = 0.004). According to these equations, at least 151 and 28 environments were required to reach the threshold of CV_*P*i,yield_ = 10% for stable and unstable genotypes, respectively. If the threshold = 5%, minimal *N*_env_ for stable and unstable genotypes is 718 and 111, respectively. This suggested that minimal *N*_env_ for robust estimation of *P*_i,yield_ is genotype and threshold dependent. Under the threshold of 10% CV_*P*i,yield_, more than 150 environments are required. We expanded this analysis to three different sampling methods and 11 SI (Supplementary Fig. S3). In general, the choice of SI, rather than the sampling method, determined the minimal required *N*_env_ for robust estimation of stability.

### More than 1000 genotypes are required to establish robust correlations between physiological parameters and yield stability

**Fig. 3:**
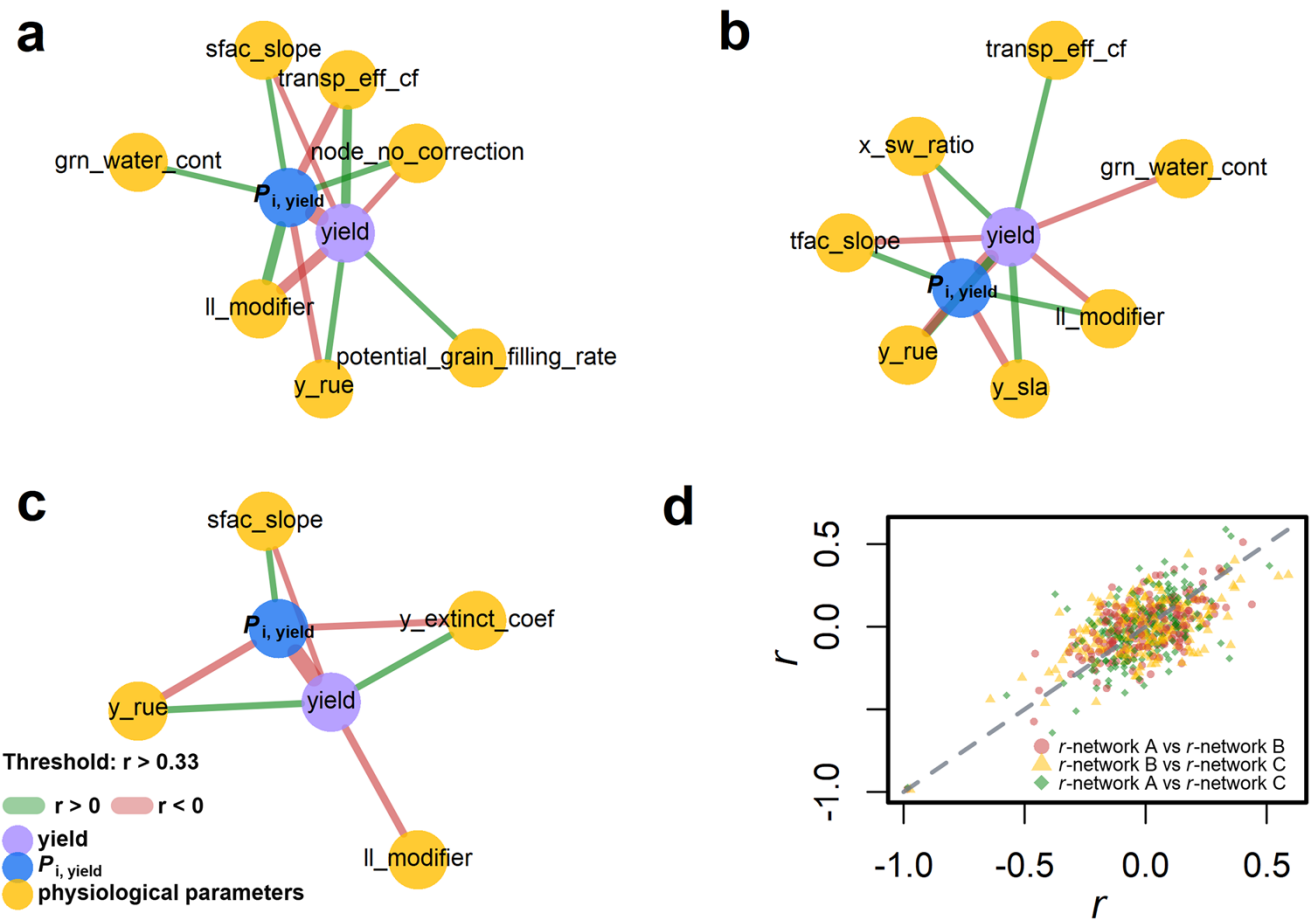
Three *r*-network of yield, *P*_i,yield_ and 90 physiological parameters from three different sampled populations of genotype (**a–c**, *N*_gen_ = 100) grown under 9000 environments (SPE = 1). Nodes (circle) represent mean yield (purple), *P*_i,yield_ (blue) or physiological parameters (yellow). Correlation between two nodes presents as a line (edge), which’s width is proportional to the absolute value of Pearson correlation coefficient (|*r*|). Green and red lines mark positive and negative *r* value, respectively. Nodes are shown with connected edge when |*r*| > 0.33. **d** Comparison of *r*-vector (Supplementary Fig. S2c) from **a–c**. Colour and shape stand for the combination of networks. Grey dashed line stands for the 1:1 line. Description of physiological parameters: water content of grain (*grn_water_cont*), number of grains that are set depending on the stem dry weight (*grain_per_gram_stem*), efficiency of roots to extract soil water (*ll_modifier*), rate of node senescence on main stem (*node_sen_rate*), potential rate of grain growth at grain filling (*potential_grain_filling_rate*), sensitivity to photoperiod (*photop_sens*), soil water effect on biomass accumulation (*sfac_slope*), transpiration efficient (*transp_eff_cf*), temperature effect on biomass accumulation (*tfac_slope*), water availability affecting the stress factor for root depth growth (*x_sw_ratio*), fraction of dry matter allocated to rachis for specific stages (*y_frac_leaf*), radiation use efficiency (*y_rue*), potential leaf specific area (*y_sla*), Extinction coefficient of green leaves as a response to row spacing (*y_extinct_coef*) (colour figure online)

To illustrate the relationship between physiological parameters, yield and *P*_i,yield_ (nodes), Pearson correlation coefficient, *r* (edges), between nodes were visualized by connecting the nodes with edges in a *r*-network (Supplementary Fig. S2c). Since the robustness of *P*_i,yield_ is related to number of environments in an exponential manner (Fig. 2c), 9000 environments (*N*_env_ = 9000, SPE = 1) were used to ensure the robustness of *P*_i,yield_ estimation. From the 9100 virtual genotypes, we first selected 100 virtual genotypes for three times (*N*_gen_ = 100, SPG = 3) using “*random*” sampling method to demonstrate the effects of genotype selection on the robustness of the edge in the *r*-network (Fig. 3).

Between three randomly selected SPG with “*random*” method, number and width (positively correlated to |*r*|) of edges and type of nodes varied between *r*-networks with a threshold of |*r*| > 0.33 for displaying the edges (Fig. 3a–c). Among these three *r*-networks, two physiological parameters related to efficiency of roots to extract soil water, linked to plant water status (*ll_modifier*, mean *r* to *P*_i,yield_ = 0.41 ± 0.15, mean *r* to yield = − 0.49 ± 0.13), potential radiation use efficiency for biomass production (*y_rue*, mean *r* to *P*_i,yield_ = − 0.48 ± 0.08, mean *r* to yield = 0.43 ± 0.08) show medium correlation to yield and *P*_i,yield_ (Supplementary Table S1). Interestingly, while *P*_i,yield_ and yield were highly negatively correlated (*r* = − 0.98, − 0.98 and − 0.97 in *r*-network a-c), a physiological parameter might tend to explain yield better than *P*_i,yield_, and vice versa. For example, the mean tendency of *ll_modifier* (*T*_*ll_modifier*_) was 0.83 ± 0.11 (SPG = 3), indicating *ll_modifier* is more correlated to yield. In contrast, the mean *T*_*y_rue*_ was 1.13 ± 0.01, indicating *y_rue* is more correlated to *P*_i,yield_.

**Fig. 4:**
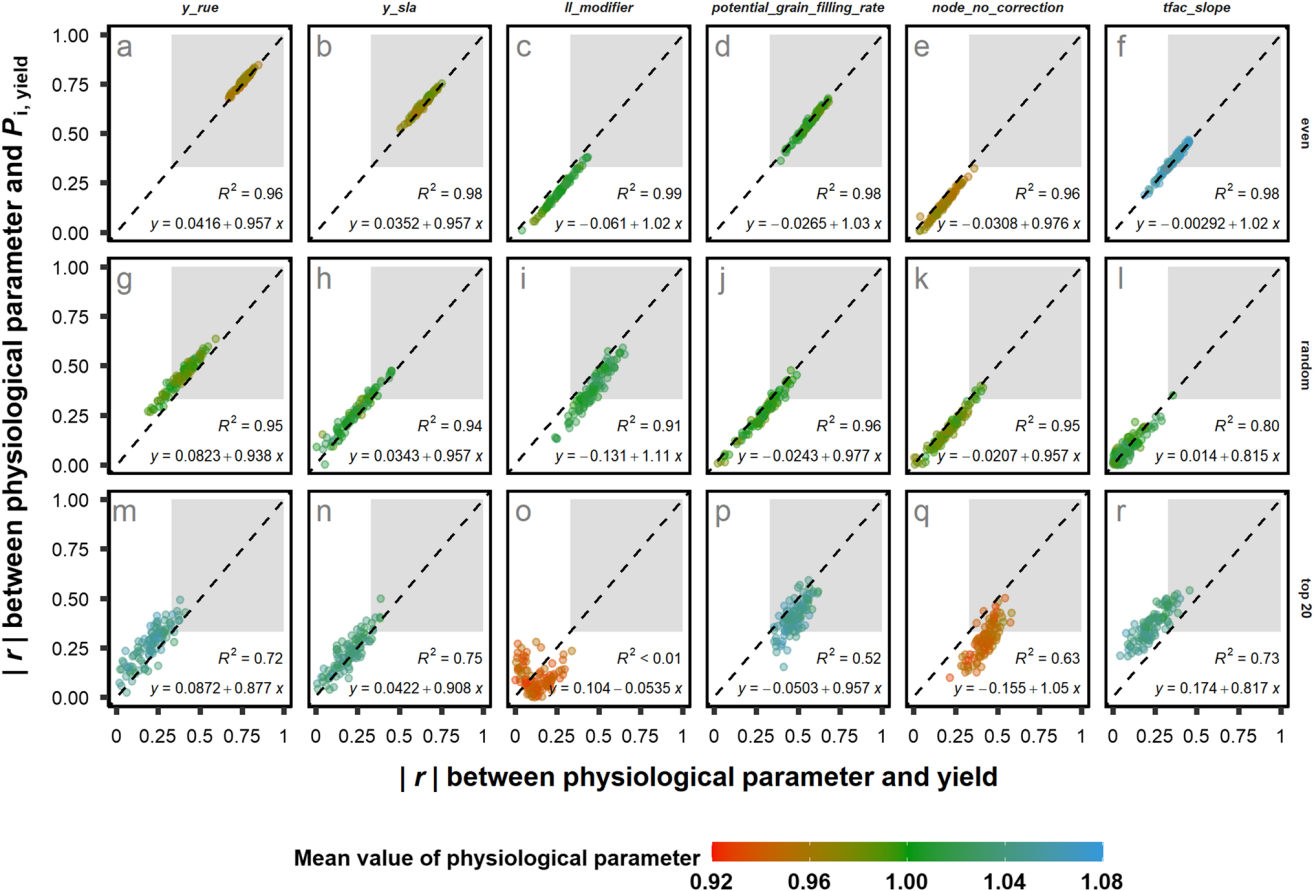
Scatterplots of |*r*| of six physiological parameters to *P*_i,yield_ versus |*r*| to yield from three selection methods (“*random*”, “*even*” and “*top 20*”) of 100 genotypes (*N*_gen_ = 100, SPG = 100) from 9000 environments (*N*_env_ = 9000, SPE = 1). Each point represents a SPG and colour of the point represents the mean values (normalized to one by the parameter values of calibrated cultivar *Hartog*) of physiological parameters from 100 genotypes in one SPG. The “*even*” sampling method divides population into ten groups based on mean yield value, with ten virtual genotypes randomly selected in each yield group. The “*top 20*” method randomly selects virtual genotypes with mean yield above 80th percentile of the whole population**.** Grey rectangle area stands for the region of |*r*|> 0.33. Black dashed line stands for 1:1 line. Meaning of the physiological parameters (in *italic*) follow the order (left to right) in the figure: radiation use efficiency (*y_rue*), potential leaf specific area (*y_sla*), efficiency of roots to extract soil water (*ll_modifier*), potential grain growth rate at grain filling (*potential_grain_filling_rate*), number of growing leaves in the sheath (*node_no_correction*) and temperature effect on biomass accumulation (*tfac_slope*)

To investigate the response of *T*_parameter_ to three sampling methods (“*random*”, “*even*” and “*top 20*”), 100 virtual genotypes (*N*_gen_ = 100) were selected for 100 times (SPG = 100) and grown under 9000 environments (*N*_env_ = 9000, SPE = 1). Six physiological parameters having the highest |*r*| with yield and with *P*_i,yield_ and with varying *T*_parameter_ were identified (Fig. 4): potential radiation use efficiency for biomass production (*y_rue,* g MJ^−1^); potential leaf specific area (*y_sla*, unitless), which determines the final leaf size; efficiency of roots to extract soil water, linked to plant water status (*ll_modifier*, unitless); potential grain growth rate at grain filling (*potential_grain_filling_rate*, g day^−1^); number of growing leaves in the sheath (*node_no_correction*, leaf), and temperature effect on biomass accumulation (*tfac_slope*, unitless). Interestingly, the range of |*r*| between physiological parameters and yield (or *P*_i,yield_) depends on the sampling method. For example, |*r*| between *y_rue* and *P*_i,yield_ was the highest (0.77 ± 0.03) from method “*even*”, followed by “*random*” (0.45 ± 0.08) and “*top 20*” (0.27 ± 0.1). In “*top 20*”, *node_no_correction*, showed the highest |*r*| with *P*_i,yield_ (0.29 ± 0.09), followed by “*random*” (0.18 ± 0.09) and “*even*” (0.15 ± 0.06). This indicates the effects of sampling method on the explanatory power of a physiological parameter for yield and *P*_i,yield_.

In contrast to a “*random*” and “*even*” SPG, the most yield and *P*_i,yield_ relevant parameter was not *y_rue* in a “*top 20”* SPG, but the parameters controlling grain filling (*potential_grain_filling_rate*), leaf expansion (*node_no_correction*) and temperature effect on biomass accumulation (*tfac_slope*). Among these six parameters, *ll_modifier*, *node_no_correction* and *potential_grain_filling* explained yield better than *P*_i,yield_. By contrast, *tfac_slope*, *y_rue* and *y_sla* explain *P*_i,yield_ better than yield (Table 1). The effects of sampling methods on *T*_parameter_ suggested that the importance of a target trait for yield or *P*_i,yield_ depends on the shape of phenotype distribution in a SPG.

### Relationships between physiological parameters and yield stability depend on the sampled population of genotypes but not affected by the population size of sampled environments

**Fig. 5:**
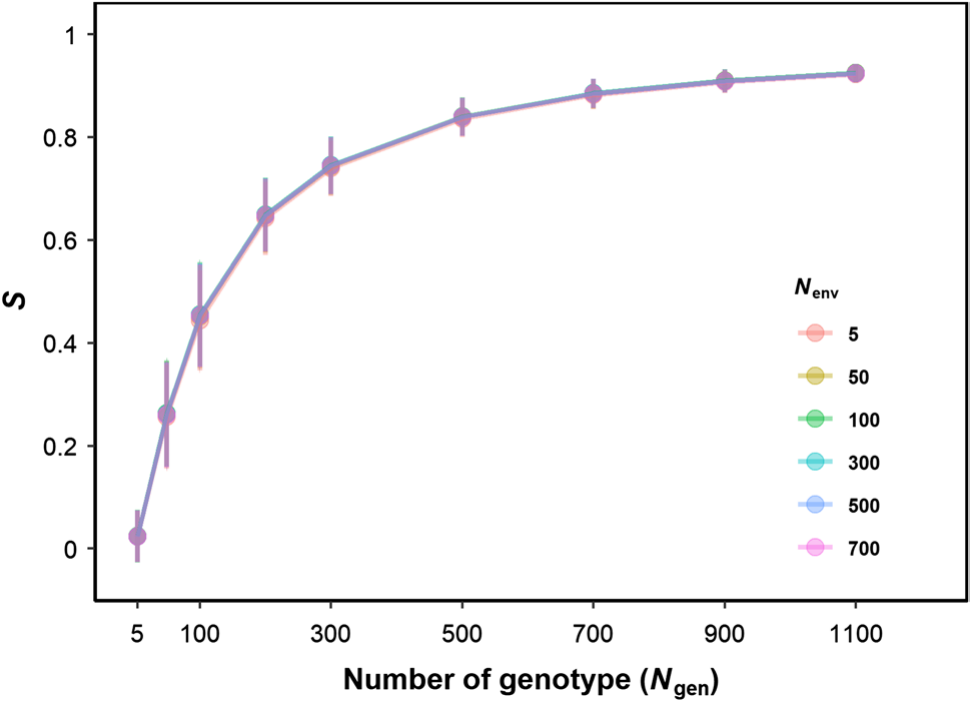
Impact of genotype number (*N*_gen_ = 5, 100, 300, 500, 700, 900 and 1100, SPG = 100) and environment number (*N*_env_ = 5, 50, 100, 300, 500 and 700, SPE = 1) on similarity (*S*) between *r*-networks

Since *r*-networks (Fig. 3) depend on the sampled genotypes in a relatively small population (*N*_gen_ = 100), proper *N*_gen_ and *N*_env_ required for a robust estimation of *r*-networks were tested using 100 SPG in combination with nine genotype numbers (*N*_gen_ = 5, 50, 100, 200, 300, 500, 700, 900 and 1100) and six environment numbers (*N*_env_ = 5, 50, 100, 300, 500 and 700). The similarity between networks (*S*) increased with *N*_gen_ but not with *N*_env_ (Fig. 5). Hence, *S* was fitted with *N*_gen_ using an asymptotic function *S* = *N*_gen_/(*k* + *N*_gen_) with *k* = 111.71 ± 5.43. Using the asymptotic function, *S* reached 0.90 with *N*_gen_ = 1006.

### Physiological network of multi-traits and their stability from random selected population

**Fig. 6:**
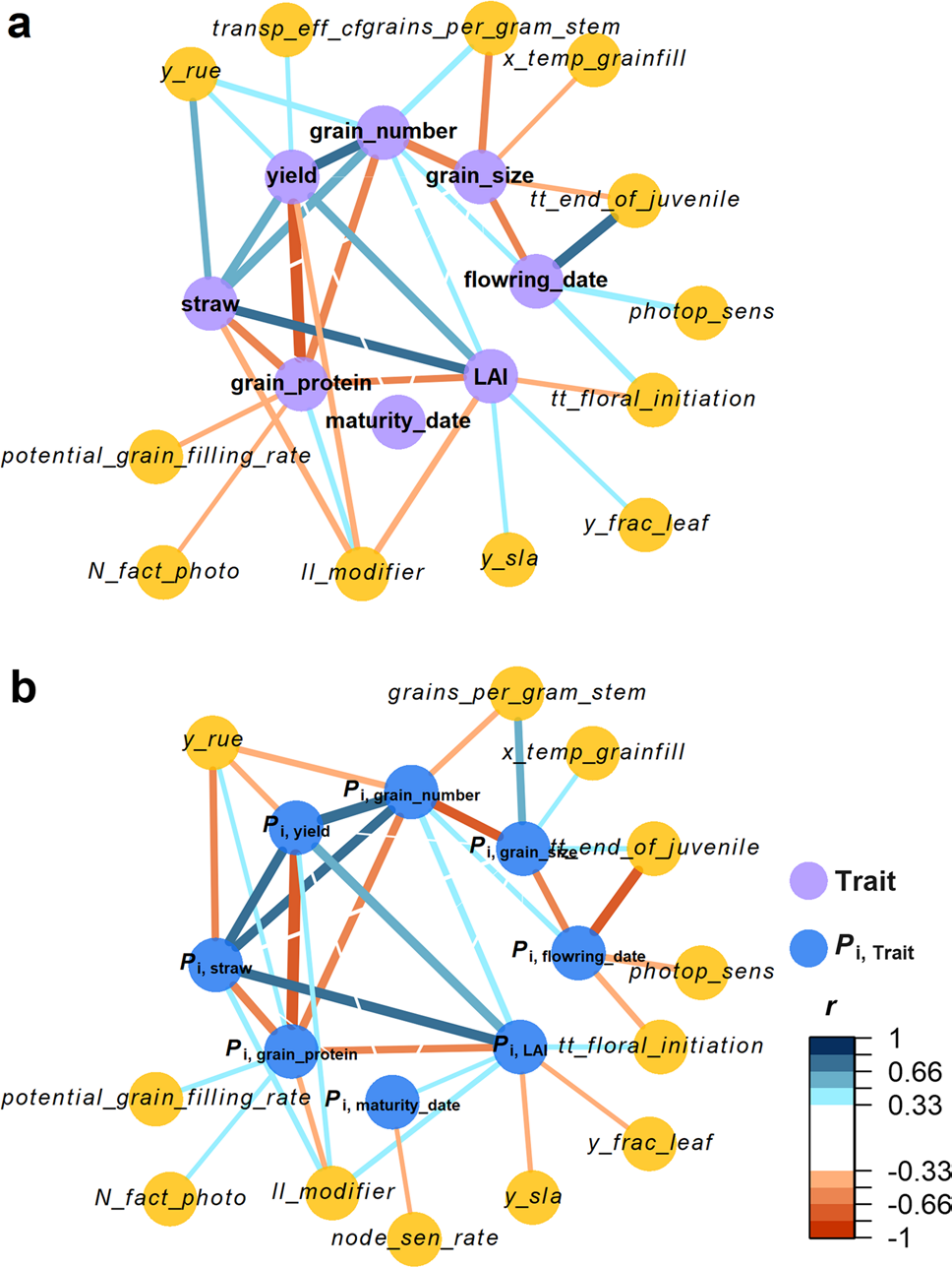
*r*-network between 90 physiological parameters, *P*_i,trait_ (**a**) and traits (**b**) of randomly selected 1000 virtual genotypes (SPG = 1) grown under 9000 environments (SPE = 1). Node (circle) represents mean trait (purple), *P*_i,yield_ (blue) or physiological parameters (yellow). Description of physiological parameters: number of grains that are set depending on the stem dry weight (*grain_per_gram_stem*), efficiency of roots to extract soil water (*ll_modifier*), rate of node senescence on main stem (*node_sen_rate*), multiplier for nitrogen deficit effect on photosynthesis (*N_fact_photo*), potential rate of grain growth at grain filling (*potential_grain_filling_rate*), sensitivity to photoperiod (*photop_sens*), transpiration efficient (*transp_eff_cf*), thermal time between plant emergence and end of juvenile stage (*tt_end_of_juvenile*), thermal time between end of juvenile and floral initiation (*tt_floral_initiation*), temperature affecting stress index for the potential grain filling rate (*x_temp_grain_fill*), fraction of dry matter allocated to rachis for specific stages (*y_frac_leaf*), radiation use efficiency (*y_rue*), potential leaf specific area (*y_sla*) (colour figure online)

Since high number of genotypes should be used to obtain robust *r*-network (Fig. 5), we conducted network analysis for physiological parameters and eight model outputs (yield, straw yield, grain protein, grain number, grain size, flowering time, maturity time and LAI) and *P*_i,Trait_ with *N*_gen_ = 1000 (SPG = 1) and *N*_env_ = 9000 (SPE = 1). Among all traits, grain number correlated most positively with yield (*r* = 0.71), followed by straw (*r* = 0.64) and LAI (*r* = 0.60). By contrast, grain protein correlated most negatively with yield (*r* = − 0.83, Fig. 6a). In the same vein, *P*_i,grain_number_ correlated most positively with *P*_i,yield_ (*r* = 0.78), followed by *P*_i,straw_ (*r* = 0.69) and *P*_i,LAI_ (*r* = 0.65) and *P*_i,grain_protein_ negatively correlated with *P*_i,yield_ (*r* = − 0.82). In general, correlations between *P*_i,Trait_, were slightly higher than that between traits (Fig. 6 and Supplementary Table S2).

Grain yield was mostly explained by efficiency of roots to extract soil water (*ll_modifier* to yield, *r* = − 0.44, *T*_*ll_modifier*_ = 0.81), while the variations in *P*_i,yield_ were best explained by radiation use efficiency (*y_rue* to *P*_i,yield_, *r* = 0.36, *T*_*y_rue*_ = 1.15). Thermal time between plant emergence and end of juvenile stage (*tt_end_of_juvenile*) most correlated to flowering time and explained this trait and its stability equally (*tt_end_of_juvenile* to flowering_date, *r* = 0.73, *T*_*tt_end_of_juvenile*_ = 1). Meanwhile, this physiological parameter also correlated with grain weight (*tt_end_of_juvenile* to grain_size, *r* = − 0.37, *T*_*tt_end_of_juvenile*_ = 0.98). LAI negatively correlated with thermal time before floral initiation (*tt_floral_initiation* to LAI, *r* = − 0.39, *T*_tt_floral_initiation_ = 0.95) and efficiency of roots to extract soil water (*ll_modifier* to LAI, *r* = − 0.46, *T*_ll_modifier=_0.87), while positively correlated with fraction of dry matter allocated to rachis for specific stage (*y_frac_leaf* to LAI, *r* = 0.34, *T*_y_frac_leaf_ = 1.05) and potential leaf specific area (*y_sla* to LAI, *r* = 0.34, *T*_y_sla_ = 1.07). The edge of *r*-networks in Fig. 6 can be found in Supplementary Table S3.

## Discussion

### Using R package *toolStability* as a tool for reproducible analysis

To study yield stability systematically, we developed and shared an *R* package *toolStability* to analyse a virtual dataset containing ~ 82 million simulation outputs obtained from the APSIM-Wheat crop model. Our *R* package *toolStability* provides more indices in comparison with other published *R* packages (Branco 2015; Ajay et al. 2018; Yaseen et al. 2018) and online tool platform (Pour-Aboughadareh et al. 2019). Furthermore, *toolStability* adds genotypic superiority measure (*P*_i,yield_, Lin and Binns 1988), a stability index which was not implemented before. While the characteristic of different SI and their pairwise correlations have been studied and reviewed in the past (Fasahat 2015; Mohammadi and Amri 2008; Piepho and Lotito 1992), there is no consensus in favour of a representative SI (Reckling et al 2021). The main reason is due to each SI has its assumption and limitation (Lin et al. 1986; Becker and Léon 1988). For example, parametric SI has the advantage of using model that is easy for implementation and interpretation, while it is poor at describing the multivariate phenotypic response to environment or having risk of misleading when the assumption is wrong. Nonparametric SI can bypass this problem of parametric methods, while reference genotype may be required to compare genotype ranking. Multivariate methods are useful in finding extreme genotypes in phenotypic stability but usually hard to interpret. Here we want to emphasize that *P*_i,yield_ was chosen in this study because it is an index characterizing high and stable yield at the same time, regardless of the population distribution of yield and among all 11 SI (Supplementary Fig. S1). Despite concerns about *P*_i_ (Fasahat 2015; Purchase et al. 2000), *P*_i_ is still useful for field studies (Mohammadi and Amri 2008; Sehgal et al. 2017) and suitable to demonstrate our analyses.

To calculate a stability index of a genotype, a population of genotypes and environments is always required (Tollenaar and Lee 2002; Sehgal et al. 2017). Therefore, it is essential to know how many genotypes and environments are necessary for an accurate estimation of a stability index and how the composition of a sampled population affects the SI of a genotype. This question can be only answered by investigating systematically with a substantial number of genotypes and environments, which is experimentally difficult. Crop model can fulfil this requirement by simulating large numbers of genotypes, environments and their combinations (Casadebaig et al. 2016; Senapati and Semenov 2020). It has been suggested that more than 200 environments are required if the threshold of CV_S__2__xi,yield_ is 10% (Piepho 1998). Their estimation was based on the assumption that this sample follows the scaled chi-squared distribution (Searle et al. 2010). In comparison to our simulation result (Supplementary Fig. S3a), only less than 50 environments is needed for reaching 10% of CV_S__2__xi,yield_ for all three sampling methods. Under random sampling method, more than 150 environments were required to obtain robust estimation of yield stability *P*_i,yield_ of two extreme genotypes (Fig. 2), indicating that the number of genotypes and environments in the published field trials for yield stability are insufficient (Wang et al. 2015; Sehgal et al. 2017; Voss-Fels et al. 2019). Considering this, in silico approaches could be used to assist breeding programs and pinpoint candidate mechanisms to be tested in the real world.

### Target traits for yield stability depend on the types of breeding program

To our knowledge, this is the first study that brings the shape of phenotype distributions (the distribution of genotypic means of a trait in a SPG) into the context of analysing yield stability (Fig. 1), including the minimal number of environment and genotype (Fig. 2, Supplementary Fig. S3) and the relationship between physiological parameter to yield and yield stability (Fig. 4). An interesting finding from our analysis is the effect of sampling methods on the relationship between the trait and *P*_i,Trait_ (e.g. for grain yield, Fig. 1 and Fig. 4) and the *r*-network between them and physiological parameters (Fig. 4), suggesting the differences in target traits between breeding programs. Our sampling methods (“*even*”, “*random*” and “*top 20*”) present three common shapes of phenotype distributions in genetic pools used in breeding programs.

Based on the central limit theorem (Laplace 1812), when the random sampling (method ‘*random*’) in combination with a large population size, a trait (e.g. yield) response will follow normal distribution (Juliana et al. 2020, Fig. 1a and Supplementary Fig. S4). Heterogeneous genetic background and a wide range of trait response make the “*random*” population (e.g. segregation population or evolutionary population) valuable for selecting favourable traits (Dwivedi et al. 2016; Bocci et al. 2020). In our simulation results, grain yield and *P*_i,yield_ in random population distributes normally as expected (Supplementary Fig. S4u and v). However, the distributions of the most influential physiological parameters in random populations are relatively flat (Supplementary Fig. S4g–i), implying the random combinations of evenly distributed physiological parameters might create a normal distribution of a complex trait (i.e. grain yield, similar to the method “*even*”). In contrast, trends and peaks can be observed in the distributions of parameters in “*top 20*” populations (Supplementary Fig. S4m–r).

Our "*top 20*” method represents the elite population with a high mean yield (Longin and Reif 2014; Ovenden et al. 2017). Compared with the random population, the elite population has a narrower and more homogeneous genetic background. Elite lines from the elite population are the result of selection methods like tail selection (Rebetzke et al. 2012) or recurrent selection (Vishwakarma et al. 2014; Rembe et al. 2019). Therefore, many traits of elite population are already optimized, for example, harvest index (HI; Zhu et al. 2010), nitrogen uptake (Cormier et al. 2013), or light interception (Rose and Kage 2019). The observation of optimized traits in elite population probably explains the observed effects of sampling methods on the correlations between *ll_modifier*, yield and *P*_i,yield_ (Fig. 4c, i and o). Among three sampling methods, “*top 20*” has the lowest mean |*r*| (*R*^2^ < 0.01, Fig. 4o), suggesting that it is the parameter which has been optimized in the “*top 20*” population (see the distribution of *ll_modifier* in Supplementary Fig. 4c). Our results further suggested that potential grain growth rate at grain filling (*potential_grain_filling_rate*, Fig. 4p), number of growing leaves in the sheath (*node_no_correction*, Fig. 4q) and temperature effect on biomass accumulation (*tfac_slope*, Fig. 4r) could be the target traits for further improving elite cultivars.

Even distribution of traits can be found at the early stage of the breeding program (Breseghello et al. 2009) or in certain environment conditions (Mathews et al. 2007; Voss-Fels et al. 2019). Our results suggested that a physiological parameter in an “*even*” or a “*random*” population explains yield and *P*_i,yield_ more equally (*T* close to one) and closely (*R*^2^ close to one) than in an elite population (Fig. 4 and Table 1). Therefore, if a breeder selects a physiological parameter for yield in “*even*” and “*random*” population, *P*_i,yield_ is also selected, while this is not guaranteed in an elite population. Our results emphasize that the shape of phenotype distribution is an important aspect in selecting target traits for improving yield stability.

### Insights from physiological networks regulating stability

In APSIM-Wheat crop model, the interactions between physiological parameters, environment and crop management on canopy development (leaf area index, LAI), flowering time, grain yield, grain size and grain number were predicted as a function of physiological assumptions of the model. The simulated dataset provides us a chance to glance at the contour of the complex physiological network and its relation to the shape of phenotype distributions (Fig. 4 and Supplementary Fig. S4). Whereas, for the complex trait like grain yield, it is difficult to decipher the genetic and physiological regulations due to pleiotropic effect of genes and the minor contribution of each quantitative trait gene (Schulthess et al. 2017; Parent et al. 2018). Our model analyses suggested that the efficiency of roots to extract soil water (*ll_modifier*) and radiation use efficiency (*y_rue*) have the highest correlations with yield and yield stability in the random population. Although it is not especially surprising the close relation between root water extractability (*ll_modifier*) and yield from an eco-physiological view (Richards et al. 2010; Thorup-Kristensen et al. 2020), it is surprising that the explanatory power of root water extractability is higher for yield than for yield stability (*T* < 1), which was similar to the parameter “*potential_grain_filling_rate*” (Fig. 4j). In contrast, the explanatory power of radiation use efficiency (*y_rue*, Fig. 4g) is higher for yield stability than for yield (*T* > 1), which was similar to the parameter “*y_sla*” (Fig. 4h). ‬‬‬This provides the first empirical proof that, despite of high correlation between mean yield and genotypic superiority measure (Fig. 1d), genetic and physiological regulations between them can still be different, as proposed in the previous genome-wide association study on yield stability (Sehgal et al. 2017). Our results from the model analysis showed the merits of in silico approach in associating physiological parameters differentially to closely related traits like yield and genotypic superiority measure for breeding programs (Hammer et al. 2019; Cooper et al. 2021).

The network between physiological parameters, model outputs and their stability (Fig. 6) suggests following physiological mechanisms regulating yield stability. Well-known mechanisms, including the trade-off between grain yield and grain protein (Slafer et al. 2014; Asseng et al. 2019) and the trade-off between grain number and grain size (Lichthardt et al. 2020; Voss-Fels et al. 2019), can be confirmed. Although the high correlation between model outputs (e.g. grain protein content and grain yield) is not always observed in the empirical datasets (Oury et al. 2003), a *R*^2^ of 0.6 has been reported (Lollato and Edwards 2015; Voss-Fels et al. 2019). Highly positive correlations between the stability of LAI, straw yield and grain number in the *r*-network (Fig. 6b) suggested that stable canopy development during the vegetative phase ensures sufficient pre-anthesis nitrogen reserves for grain filling and thereby yield stability. Physiologically, stable and vigorous canopy development ensures radiation interception (Tian et al. 2011) and allows storage of nitrogen and water-soluble carbohydrates in the canopy at the end of the vegetative phase (referred to as pre-anthesis nitrogen and carbon reserves, respectively).

The pre-anthesis nitrogen and carbon reserves might contribute significantly to grain filling since wheat accumulates about 70% of the total biomass and takes up about 70–100% of total nitrogen before anthesis (Barraclough et al. 2014; Wu et al. 2016). Under optimal nitrogen supply, the pre-anthesis nitrogen reserves in stems, sheathes and leaves contribute about 30%, 15% and 40% of the nitrogen content in wheat grains, respectively (re-calculated from Fig. 3 of Barraclough et al. 2014). These results indicate the importance of pre-anthesis nitrogen reserves on grain yield. Although forty years ago, the estimated contribution of pre-anthesis carbon reserves to grain weight ranged between 11 and 17% but is higher under stress conditions (up to 22–44%) due to the lower yield level. Since genetic variation of pre-anthesis carbon reserves in wheat exists (Ehdaie et al. 2006), together with the modern wheat cultivars have higher pre-anthesis carbon reserves than the old cultivars (Xiao et al. 2012), it is worth a revisit of the contribution of pre-anthesis carbon reserve to yield in the modern cultivars.

Deriving from the data of a recent study using 20 wheat cultivars suggests that, on average, biomass accumulation before anthesis may contribute up to 38–43% of the grain yield (Barraclough et al. 2014). High contribution to grain yield from pre-anthesis reserves indicates the potential role of pre-anthesis carbon reserve as a buffer to secure the yield. In other words, yield stability could be achieved by increasing the pre-anthesis carbon reserve pool that reduces the risk of insufficient photosynthate at the grain filling stage due to abiotic stress (Slewinski 2012). This also explains the early observation that a wheat genotype with higher biomass accumulation until anthesis, a proxy of higher pre-anthesis nitrogen and carbon reserves, has a higher yield and less yield variation between experimental years (Damisch and Wiberg 1991). Furthermore, the size of the pre-anthesis carbon reserve pool is determined by carbon fixation, namely canopy photosynthesis, during the vegetative phase, as suggested by the correlations of radiation use efficiency (*y_rue*) with *P*_i,yield_ and *P*_i,straw_ (Fig. 6). Our *r*-network also suggests close relationship between stable canopy development (low *P*_i,LAI_) and stable grain number (low *P*_i,grain_number_), probably due to the effects of canopy condition at pre-anthesis stage on floral formation (Stockman et al. 1983) or carbon and nitrogen reserves that avoid pre-anthesis abortion (Sinclair and Jamieson 2008).

Physiologically, it is noteworthy that not all traits (physiological parameters) have robust contributions to yield and yield stability and their contributions can be environment-dependent (Ferrante et al. 2017; Slafer et al. 2022). However, there are also traits (e.g. reproductive, phenological, photosynthetic and architectural traits) delivering stable and positive effects to yield formation and their contributions to yield are less environment-dependent (Welcker et al. 2022). To our opinion, these can be the traits showing significance within the network of yield and yield stability (Fig. 6; e.g. grain number, photoperiodic sensitivity and radiation use efficiency), as shown in the experimental findings in wheat (Voss-Fels et al. 2019; Lichthardt et al. 2020) and in maize (Welcker et al. 2022) that these traits with stable effects on yield have been indirectly preferred under breeders´ selections. Welcker et al. (2022) also clearly showed that physiological traits with different effects on yield between environments are phenotypically unchanged by selection. Therefore, we could speculate that the parameters showing importance in Fig. 6 are the parameters delivering stable effects on yield and can be the first target for breeders.

## Supplementary Information

Below is the link to the electronic supplementary material.

Supplementary file1 (PDF 763 KB)

## Data Availability

All data supporting the findings of this study are available within the paper and within its supplementary data published online. An *R* package *toolStability* was published on CRAN (https://cran.r-project.org/web/packages/toolStability/index.html) and Zenodo (10.5281/zenodo.5804212). An APSIM-Wheat dataset is available on Zenodo (https://doi.org/10.5281/zenodo.4729636). A repository for reproducing the figures in this publication is available on GitHub (https://github.com/Illustratien/Wang_2023_TAAG) and Zenodo (10.5281/zenodo.7562420).

## Abbreviations

APSIM: Agricultural production systems sIMulator
GxE: Genotype by environment interaction
LAI: Leaf area index
SI: Stability index
SPG: Sampled population of genotypes
SPE: Sampled population of environments
TPE: Target population of environments
