# Supplementary material for: More than 1000 genotypes are required to derive robust relationships between yield, yield stability and physiological parameters: a computational study on wheat crop
Source: Theor Appl Genet. 2023 Mar 10;136(3):34. doi: 10.1007/s00122-023-04264-7 (PMC10006026; doi:10.1007/s00122-023-04264-7)
Supplement: Supplementary file 1 — Supplementary file1 (PDF 763 KB) [file 122_2023_4264_MOESM1_ESM.pdf]

Theoretical and Applied Genetics

More than 1000 genotypes are required to derive robust relationships between yield, yield stability and physiological parameters: a computational study on wheat crop

Tien-Cheng Wang[12*], Pierre Casadebaig[3], Tsu-Wei Chen[1]

[1] Section of Intensive Plant Food Systems, Albrecht Daniel Thaer-Institute of Agricultural and Horticultural Sciences, Humboldt Universität zu Berlin, Germany; [2] Institut für Gartenbauliche Produktionssysteme, Leibniz Universität Hannover, Germany; [3] INRA, UMR1248 AGIR, Castanet-Tolosan, France.

Corresponding author

Correspondence to Tien-Cheng Wang

Email: wangtien@student.hu-berlin.de

ORCID: 0000-0001-7952-1404

Supplemental tables

Table S1. Pearson correlation coefficient (*r*, edge) between two nodes (yield, $P_{i,yield}$ or physiological parameters) in *r*-networks (Fig. 3 a-c).

node1	node2	edge(*r*)	*r*-network
yield	$P_{i,yield}$	-0.98	a
yield	*ll_modifier*	-0.64	a
$P_{i,yield}$	*ll_modifier*	0.59	a
yield	*transp_eff_cf*	0.55	a
$P_{i,yield}$	*transp_eff_cf*	-0.51	a
$P_{i,yield}$	*y_rue*	-0.42	a
yield	*node_no_correction*	-0.37	a
yield	*y_rue*	0.37	a
$P_{i,yield}$	*node_no_correction*	0.36	a
yield	*potential_grain_filling_rate*	0.34	a
$P_{i,yield}$	*sfac_slope*	0.34	a
yield	*sfac_slope*	-0.34	a
$P_{i,yield}$	*grn_water_cont*	0.33	a
yield	$P_{i,yield}$	-0.98	b
$P_{i,yield}$	*y_rue*	-0.57	b
yield	*y_rue*	0.51	b
$P_{i,yield}$	*y_sla*	-0.46	b
yield	*y_sla*	0.44	b
yield	*ll_modifier*	-0.39	b

**node1**	**node2**	**edge(*r*)**	***r*-network**
$P_{i,}$yield	*x_sw_ratio*	-0.37	b
yield	*grn_water_cont*	-0.36	b
yield	*tfac_slope*	-0.36	b
$P_{i,yield}$	*tfac_slope*	0.35	b
yield	*transp_eff_cf*	0.35	b
yield	*x_sw_ratio*	0.35	b
$P_{i,yield}$	*ll_modifier*	0.33	b
yield	$P_{i,yield}$	-0.97	c
$P_{i,yield}$	*y_rue*	-0.46	c
yield	*ll_modifier*	-0.44	c
$P_{i,yield}$	*y_extinct_coef*	-0.40	c
yield	*y_rue*	0.40	c
yield	*y_extinct_coef*	0.39	c
$P_{i,yield}$	*sfac_slope*	0.37	c
yield	*sfac_slope*	-0.34	c

Capital letters (a-c) refer to different sample population of genotypes (SPG) of different *r*-networks. Description of physiological parameters: water content of grain (*grn_water_cont*), number of grains that are set depending on the stem dry weight (*grain_per_gram_stem*), efficiency of roots to extract soil water (*ll_modifier*), rate of node senescence on main stem (*node_sen_rate*), potential rate of grain growth at grain filling (*potential_grain_filling_rate*), sensitivity to photoperiod (*photop_sens*), soil water effect on accumulation off biomass (*sfac_slope*), transpiration efficiency (*transp_eff_cf*), temperature effect on accumulation of biomass (*tfac_slope*),water availability affecting the stress factor for root depth growth (*x_sw_ratio*), fraction of dry matter allocated to rachis for specific stages (*y_frac_leaf*), potential radiation use efficiency for biomass production (*y_rue*), potential leaf specific area (*y_sla*), Extinction coefficient of green leaves as a response to row spacing (*y_extinct_coef*).

Table S2. Pearson correlation coefficient (*r*) of two nodes between simulated traits (grain yields, straw, grain size, grain number, grain protein, leaf area index (LAI), maturity date and flowering date) and stability of simulated traits ($P_{i,Trait}$) in *r*-network (Fig. 6).

**node1**	**node2**	***r***
$P_{i,yield}$	$P_{i,grain\_number}$	0.78
yield	grain_number	0.71
$P_{i,yield}$	$P_{i,LAI}$	0.65
yield	LAI	0.60
yield	maturity_date	0.22
$P_{i,yield}$	$P_{i,maturity\_date}$	0.21
$P_{i,yield}$	$P_{i,flowering\_date}$	0.13
yield	flowering_date	0.05
yield	grain_size	0.02
$P_{i,yield}$	$P_{i,grain\_size}$	-0.14
$P_{i,yield}$	$P_{i,grain\_protein}$	-0.82
yield	grain_protein	-0.83
$P_{i,straw}$	$P_{i,LAI}$	0.71
$P_{i,straw}$	$P_{i,yield}$	0.69
$P_{i,straw}$	$P_{i,grain\_number}$	0.69
straw	LAI	0.67
straw	grain_number	0.66
straw	yield	0.64
straw	maturity_date	0.13

node1	node2	*r*
$P_{i,straw}$	$P_{i,maturity\_date}$	0.13
$P_{i,straw}$	$P_{i,flowering\_date}$	0.12
straw	flowering_date	0.09
straw	grain_size	-0.27
$P_{i,straw}$	$P_{i,grain\_size}$	-0.29
straw	grain_protein	-0.60
$P_{i,straw}$	$P_{i,grain\_protein}$	-0.61
$P_{i,maturity\_date}$	$P_{i,LAI}$	0.33
maturity_date	LAI	0.33
maturity_date	grain_size	0.22
$P_{i,maturity\_date}$	$P_{i,grain\_size}$	0.21
maturity_date	grain_number	0.01
$P_{i,maturity\_date}$	$P_{i,grain\_number}$	0.00
maturity_date	grain_protein	-0.12
$P_{i,maturity\_date}$	$P_{i,grain\_protein}$	-0.15
maturity_date	flowering_date	-0.21
$P_{i,maturity\_date}$	$P_{i,flowering\_date}$	-0.24
$P_{i,LAI}$	$P_{i,grain\_number}$	0.47
LAI	grain_number	0.39
LAI	grain_size	0.04
$P_{i,LAI}$	$P_{i,grain\_size}$	-0.05
$P_{i,LAI}$	$P_{i,flowering\_date}$	-0.10

node1	node2	*r*
LAI	flowering_date	-0.19
LAI	grain_protein	-0.50
$P_{i,LAI}$	$P_{i,grain\_protein}$	-0.53
grain_size	grain_protein	-0.03
$P_{i,grain\_size}$	$P_{i,grain\_protein}$	-0.04
grain_size	flowering_date	-0.53
$P_{i,grain\_size}$	$P_{i,flowering\_date}$	-0.53
grain_size	grain_number	-0.66
$P_{i,grain\_size}$	$P_{i,grain\_number}$	-0.69
$P_{i,grain\_protein}$	$P_{i,flowering\_date}$	-0.03
grain_protein	flowering_date	-0.04
grain_protein	grain_number	-0.59
$P_{i,grain\_protein}$	$P_{i,grain\_number}$	-0.60
$P_{i,grain\_number}$	$P_{i,flowering\_date}$	0.40
grain_number	flowering_date	0.37

Table S3. Pearson correlation coefficient (*r*, edge) and tendency ($T_{parameter}$) of one physiological parameter (in *italic*) to simulated traits (grain yields, straw, grain size, grain number, grain protein, leaf area index (LAI), maturity date and flowering date) and stability of traits ($P_{i,Trait}$) in *r*-network (Fig. 6).

Physiological parameters	Trait	***r* to $P_{i,Trait}$**	***r* to Trait**	**$T_{parameter}$**
ll_modifier	yield	0.36	-0.44	0.81
transp_eff_cf	yield	-0.30	0.33	0.91
y_rue	yield	-0.42	0.36	1.15
ll_modifier	straw	0.42	-0.48	0.87
y_rue	straw	-0.53	0.51	1.04
node_sen_rate	maturity_date	-0.33	0.32	1.04
ll_modifier	LAI	0.40	-0.46	0.87
tt_floral_initiation	LAI	0.37	-0.39	0.95
y_frac_leaf	LAI	-0.35	0.34	1.05
y_sla	LAI	-0.37	0.34	1.07
grains_per_gram_stem	grain_size	0.56	-0.54	1.03
tt_end_of_juvenile	grain_size	0.37	-0.37	0.98
x_temp_grainfill	grain_size	0.34	-0.34	0.99
ll_modifier	grain_protein	-0.40	0.42	0.96
N_fact_photo	grain_protein	0.34	-0.33	1.02
potential_grain_filling_rate	grain_protein	0.37	-0.38	0.97
y_rue	grain_protein	0.35	-0.32	1.10
grains_per_gram_stem	grain_number	-0.43	0.45	0.94

**Physiological parameters**	**Trait**	***r* to $P_{i,\mathrm{Trait}}$**	***r* to Trait**	**$T_{\mathrm{parameter}}$**
y_rue	grain_number	-0.47	0.45	1.04
photop_sens	flowering date	-0.49	0.48	1.02
tt_end_of_juvenile	flowering date	-0.73	0.73	1.00
tt_floral_initiation	flowering date	-0.44	0.45	0.98

Description of physiological parameters: number of grains that are set depending on the stem dry weight (*grain_per_gram_stem*), efficiency of roots to extract soil water (*ll_modifier*), rate of node senescence on main stem (*node_sen_rate*), multiplier for nitrogen deficit effect on photosynthesis (*N_fact_photo*), potential rate of grain growth at grain filling (*potential_grain_filling_rate*), sensitivity to photoperiod (*photop_sens*), transpiration efficiency (*transp_eff_cf*), thermal time between plant emergence and end of juvenile stage (*tt_end_of_juvenile*), thermal time between end of juvenile and floral initiation (*tt_floral_initiation*), temperature affecting stress index for the potential grain filling rate (*x_temp_grain_fill*), fraction of dry matter allocated to rachis for specific stages (*y_frac_leaf*), radiation use efficiency (*y_rue*), potential leaf specific area (*y_sla*).

Supplemental figures

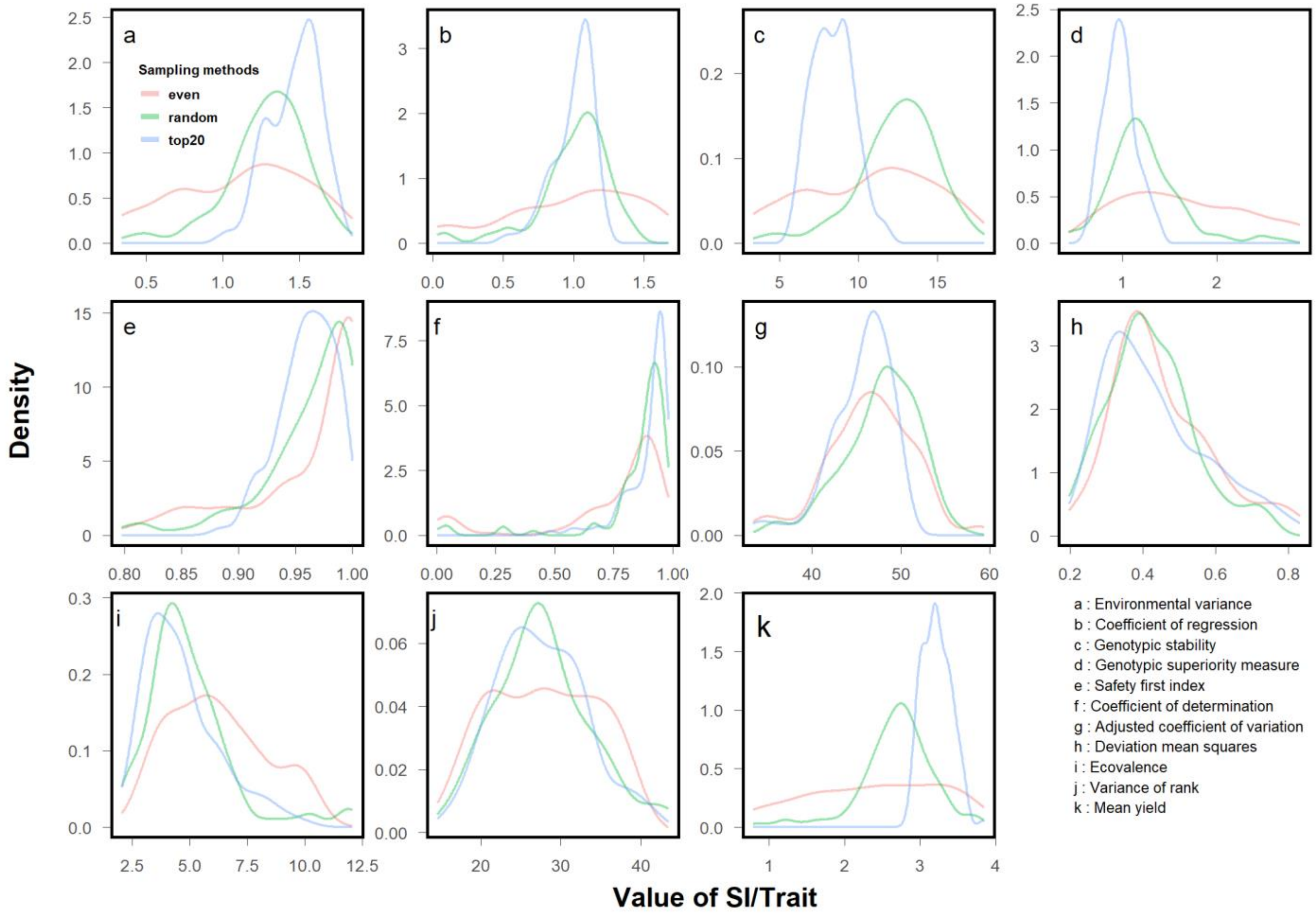

Fig. S1 Effect of three sampling methods ("*even*", "*random*", "*top 20*") on the shape of value distribution from 10 stability indices and mean yield of 100 genotypes (N_{gen}=100, SPG=100) in 100 environments (N_{env}=100, SPE=1). Three sampling methods were marked with blue, green and red for "*top 20*", "*random*" and "*even*", respectively. The ten SI are (a) environmental variance, (b) coefficient of regression, (c) genotypic stability, (d) genotypic superiority measure, (e) safety first index, (f) coefficient of determination, (g) adjusted coefficient of variation, (h) deviation mean squares, (i) ecovalence and (j) variance of rank.

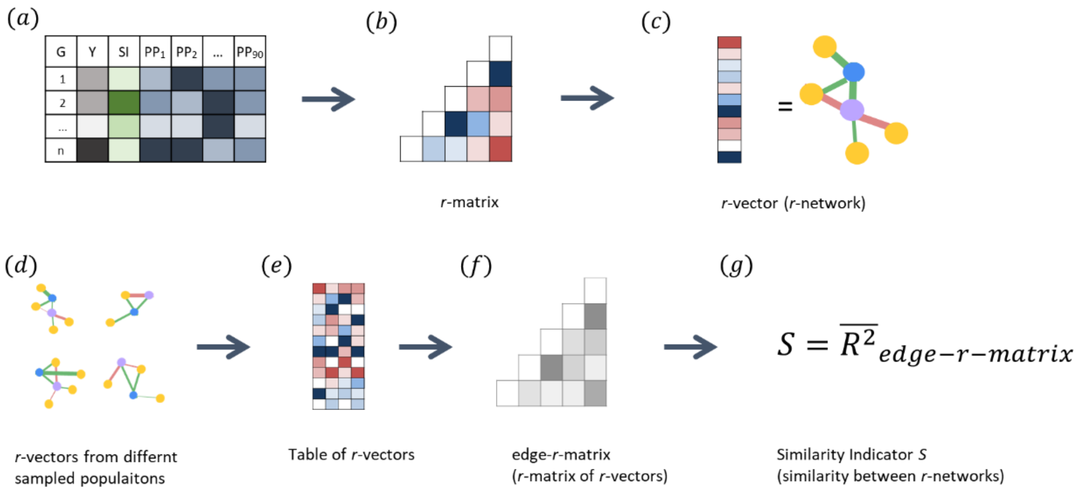

Fig. S2 Process steps for the generation of *r*-network (*r*-vector) and indicator of similarity between networks (*S*). For the network analysis of yield, yield stability and physiological parameters, first step is (a) a table of mean yield (Y), stability index (SI) and 90 physiological parameters ($PP_{1\sim90}$) for all genotypes ($G_{1\sim n}$). Second, calculate (b) correlation of coefficient matrix (*r*-matrix) from (A). Third, *r* between physiological parameters itself is excluded and resulted in (c) *r*-vector, which is later displayed as network. For the estimation of the similarity between (d) network from different SPG, First the *r*-vectors from different networks are combined into (e) a table of *r*-vectors. Second, (f) an edge-*r*-matrix is derived from calculation of correlation matrix table of *r*-vectors. Finally, (g) similarity indicator (*S*) is used as the estimation of similarity between networks by averaged of squared edge-*r*-matrix (f).

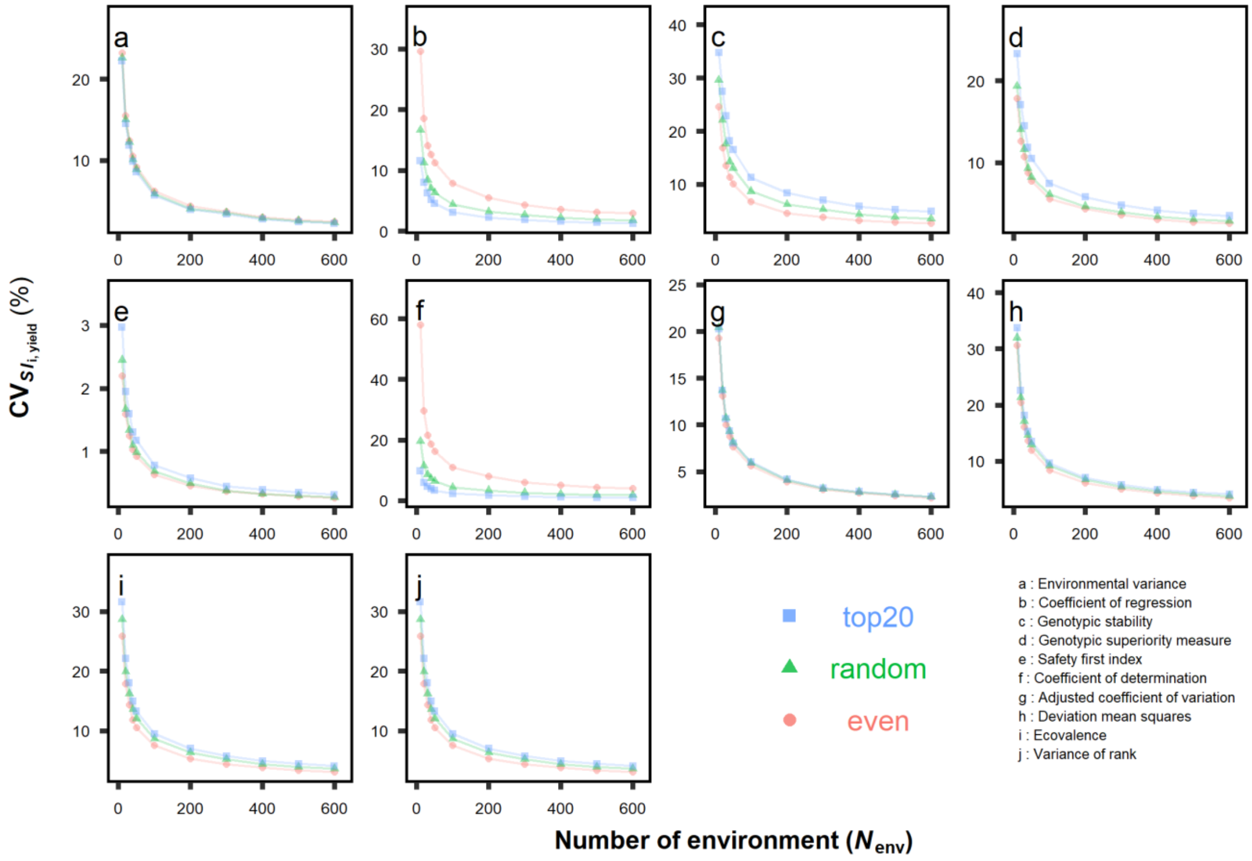

Fig. S3 Effect of three sampling methods ("*even*", "*random*", "*top 20*") on the response of coefficient of variation of stability index of yield ($CV_{SI,yield}$) between 100 SPE across number of environments (N_{env}=10-600) from 100 genotypes (N_{gen}=100, SPG = 1). Three sampling methods were marked with blue square, green triangle and red circle for "*top 20*", "*random*" and "*even*", respectively. The ten SI are (a) Environmental variance, (b) Coefficient of regression, (c) Genotypic stability, (d) Genotypic superiority measure, (e) Safety first index (f) Coefficient of determination (g) Adjusted coefficient of variation (h) Deviation mean squares (i) Ecovalence and (j) Variance of rank.

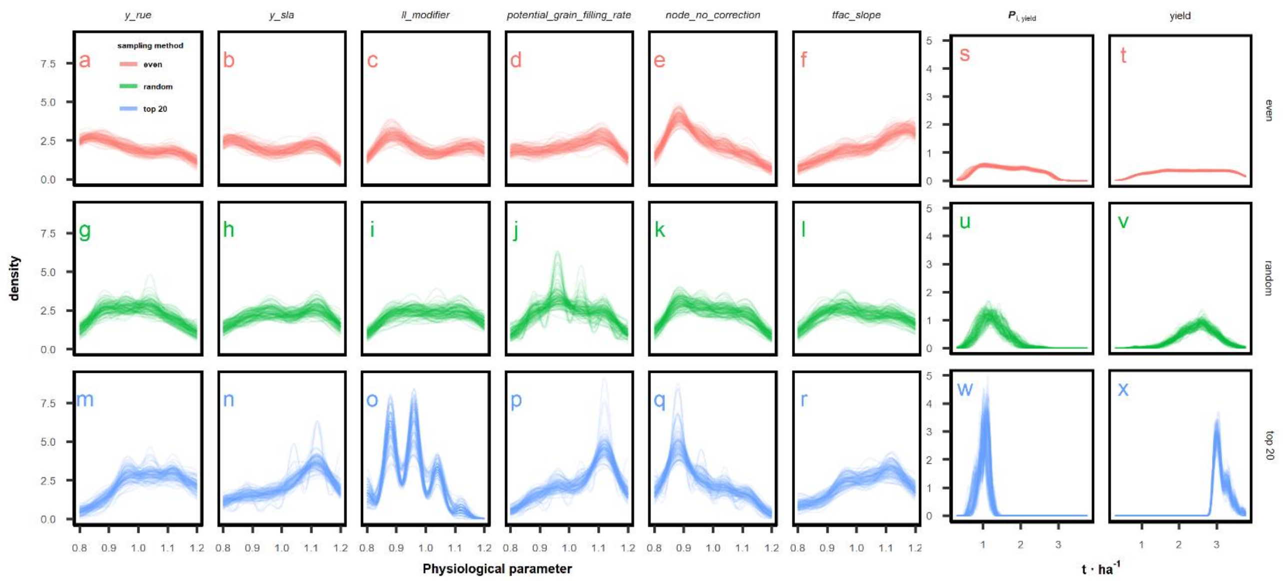

Fig. S4 Density plot for value of six physiological parameters, yield and $P_{i,yield}$ from 100 genotypes (N_{gen}=100, SPG=100) and 9000 environments (N_{env}=9000, SPE=1) with three sampling methods ("*even*", "*random*" and "*top 20*") in Fig. 4. Three sampling methods were marked with red, green and blue for "*even*", "*random*" and "*top 20*", respectively. Values of 100 genotype from same SPG were connected with one line. First 18 subplots (a-r): Value density of six physiological parameter from 100 SPG in three sampling methods. Last six subplots (s-x): yield and $P_{i,yield}$ density from 100 SPG in three sampling methods. Description of physiological parameters: potential radiation use efficiency for biomass production (*y_rue*); potential leaf specific area (*y_sla*); efficiency of roots to extract soil water (*ll_modifier*); potential grain growth rate at grain filling (*potential_grain_filling_rate*); number of growing leaves in the sheath (*node_no_correction*), and temperature effect on biomass accumulation (*tfac_slope*).
